# Cyclodextrin Alters GABAergic Input to CA1 Pyramidal Cells in Wild-Type But Not in NPC1-Deficient Mice

**DOI:** 10.1089/biores.2015.0023

**Published:** 2015-08-01

**Authors:** Moritz J. Frech, Michael Rabenstein, Katja Bovensiepen, Sebastian Rost, Arndt Rolfs

**Affiliations:** Albrecht-Kossel-Institute for Neuroregeneration, University of Rostock, Rostock, Germany.

**Keywords:** cyclodextrin, hippocampus, Niemann–Pick type C1, patch clamp, postsynaptic currents

## Abstract

Niemann–Pick type C1 disease (NPC1) is a neurodegenerative disorder caused by mutations in the *NPC1* gene. Actual, no causative treatment for NPC1 is available, although some drugs have been proven to be beneficial to patients, for example, 2-hydroxypropyl-β-cyclodextrin (CDX). In this study, we used the BALB/c_Nctr-Npc1m1N/-J mouse strain to study the effect of CDX, which is described to prolong the life span and to alleviate the pathogenic phenotype. By means of patch clamp recordings, we measured inhibitory postsynaptic currents (IPSCs) of CA1 pyramidal cells of CDX-treated and -untreated animals to elucidate the influence of CDX on the synaptic transmission. Surprisingly, CDX induced a significantly higher GABAergic IPSC frequency in wild-type mice than in NPC1^−/−^ mice. Although the IPSCs were mainly GABAergic, we observed a significant reduction of the IPSC frequency in the presence of the glycine receptor antagonist strychnine. The effect of strychnine did not differ in untreated and treated animals, indicating that the effect of CDX was most likely not based on an interaction with glycinergic transmission machinery. However, the unexpected effect of CDX on the GABAergic synaptic transmission is of special interest as a disturbance plays, for example, a crucial role in epilepsy and, moreover, as CDX is currently under investigation as a treatment for NPC1 in humans.

## Introduction

Niemann–Pick type C1 disease (NPC1) is a rare progressive neurodegenerative disease caused by mutations in the *NPC1* gene, leading to an impaired lipid transport and an accumulation of cholesterol and gangliosides in the late endosomes and lysosomes. Besides clinical manifestation like hepatosplenomegaly, seizures, dementia, and cerebellar ataxia, a progressive neurological degradation is a striking hallmark of NPC1.^1^ Although a variety of morphological alterations of neurons are described,^2,3^ the pathogenic mechanisms remain to be elucidated. Cholesterol is essential for a proper synaptic transmission, as receptor clustering depends on cholesterol^4^ as well as fusion and release of synaptic vesicles.^5,6^ A disturbance of synaptic transmission and plasticity may be causative for clinical symptoms, and thus, studies in this regard are of special interest. An altered excitatory synaptic transmission was observed in cultured hippocampal neurons from NPC1^−/−^ mice and in hippocampal slices.^7,8^ Thus, we asked if any alterations of inhibitory transmission can be found in the hippocampal CA1 formation of NPC1-deficient mice. Furthermore, we were interested in the effect of 2-hydroxypropyl-β-cyclodextrin (CDX) on the synaptic transmission, which has been proven to be beneficial in NPC1^−/−^ mice.^9^

## Materials and Methods

### Preparation of hippocampal slices and patch clamp recordings

Animals of the BALB/c_Nctr-Npc1m1N/-J strain (Jackson Laboratories) and wild-type (WT) animals were weekly injected subcutaneously with CDX starting at p7 (4 g/kg body weight, dissolved in 0.9% NaCl) as recently described.^9^ All experiments were carried out in accordance to the German Protection of Animals Law. Hippocampal slices of mice (median days of age 58, 25/75% percentile = 56/62) were prepared using the *magic cut*.^10^ Patch clamp recordings were performed at room temperature using an EPC-10 amplifier (Heka). Patch pipettes were pulled from borosilicate glass (Harvard Apparatus). Internal solution contained (mM): KCl 140, HEPES 10, EGTA 11, MgCl_2_ × 6H_2_O 1, CaCl_2_ × 2H_2_O 1, and pH 7.2. Filled electrodes had a resistance of 4–8 MΩ. Slices were superfused with extracellular solution containing (mM): NaCl 125, KCl 2.5, CaCl_2_ × H_2_O 2, MgCl_2_ × 6H_2_O 1, NaHCO_3_ 26, NaH_2_PO_4_ × H_2_O 1.25, glucose × H_2_O 25, and pH 7.3–7.4. Recordings were made in the whole-cell configuration with a holding potential of −60 mV. Data were filtered at 3 kHz and digitized with 10 kHz using Pulse 8.80 (Heka). Postsynaptic currents were analyzed with Mini Analysis 6 (Synaptosoft).

Animals per group: WT untreated = 16; WT treated = 7, NPC1^−/−^ untreated = 22, NPC1^−/−^ treated = 7. *n* indicates number of single recordings.

### Statistical analysis

Analysis was carried out with GraphPad Prism6 (GraphPad Software, Inc.). Data are given as mean ± standard error of the mean. Paired or unpaired Student's *t*-test was used to test for significance, with *p < 0.05; **p < 0.01, ***p < 0.001. *p*-Value < 0.05 was considered to indicate statistically significant differences.

## Results

### CDX impairs GABAergic transmission in WT but not in NPC1^−/−^ mice

**FIG. 1.**
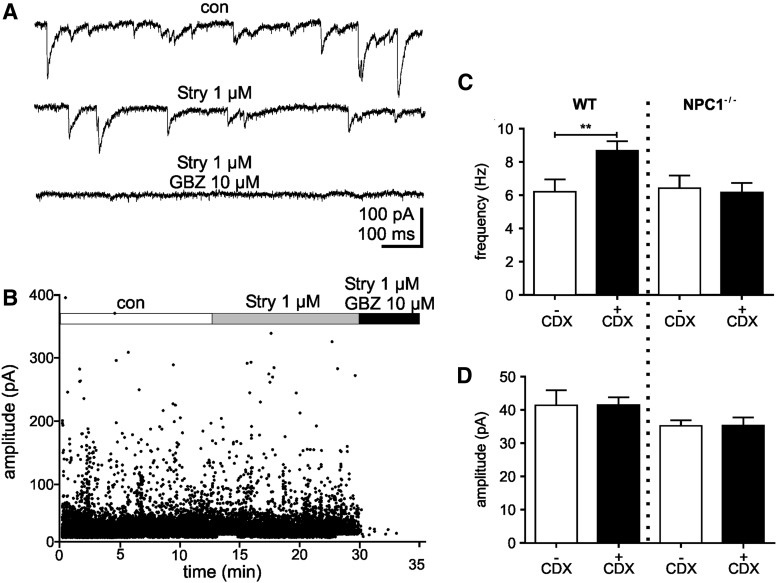
**(A)** IPSCs recorded under control, Stry, and Stry+GBZ. **(B)** Plot of IPSC amplitudes versus time. IPSCs recorded in the presence of Stry are referred as GABAergic IPSCs, as they were blocked by GBZ. Analysis of frequencies **(C)** and amplitudes **(D)** of IPSCs recorded in untreated mice (−CDX) and treated mice (+CDX) revealed significantly higher frequencies in WT mice but not in NPC1^−/−^ mice. CDX, 2-hydroxypropyl-β-cyclodextrin; GBZ, gabazine; IPSCs, inhibitory postsynaptic currents; NPC1, Niemann–Pick type C1 disease; Stry, strychnine; WT, wild type.

In this study, we measured inhibitory postsynaptic currents (IPSCs) of pyramidal cells in the CA1 region of the hippocampus by means of patch clamp recordings. Using a symmetrical Cl^−^ concentration and a holding potential of −60 mV, the activation of Cl^−^-permeable ion channels like GABA_A_ receptors (GABA_A_-Rs) or glycine receptors (Gly-Rs) was demonstrated as inward directed currents (Fig. 1A). We used the antagonists gabazine (GBZ; 5 μM) and strychnine (Stry; 1 μM) to antagonize GABA_A_-Rs- and Gly-Rs-mediated IPSCs. The application of GBZ resulted in a block of the IPSCs (Fig. 1A, B), indicating that the IPSCs were mediated by GABA_A_-Rs and not by Gly-Rs. Consequently, we did not observe IPSCs in experiments starting with an application of GBZ (data not shown). In the following, IPSCs recorded in the absence of antagonists are referred as control (con) and in the presence of strychnine as GABAergic IPSCs.

The analysis of the IPSC-frequencies (*f*) revealed comparable values under control conditions for WT and NPC1^−/−^ mice (6.2 ± 0.8 Hz, *n* = 22; 6.4 ± 0.8 Hz, *n* = 27; respectively; Fig. 1C).

Surprisingly, we observed in CDX-treated WT, but not CDX-treated NPC1^−/−^-animals, an elevated IPSC-frequency (WT/CDX: *f* = 8.7 ± 0.6 Hz, *n* = 17; NPC1^−/−^/CDX: *f* = 6.2 ± 0.6 Hz, *n* = 19; Fig. 1C), where the amplitudes did not differ (WT/CDX: 41.5 ± 2.3 pA, *n* = 17; NPC1^−/−^/CDX: 35.3 ± 2.5 pA, *n* = 19; Fig. 1D).

**FIG. 2.**
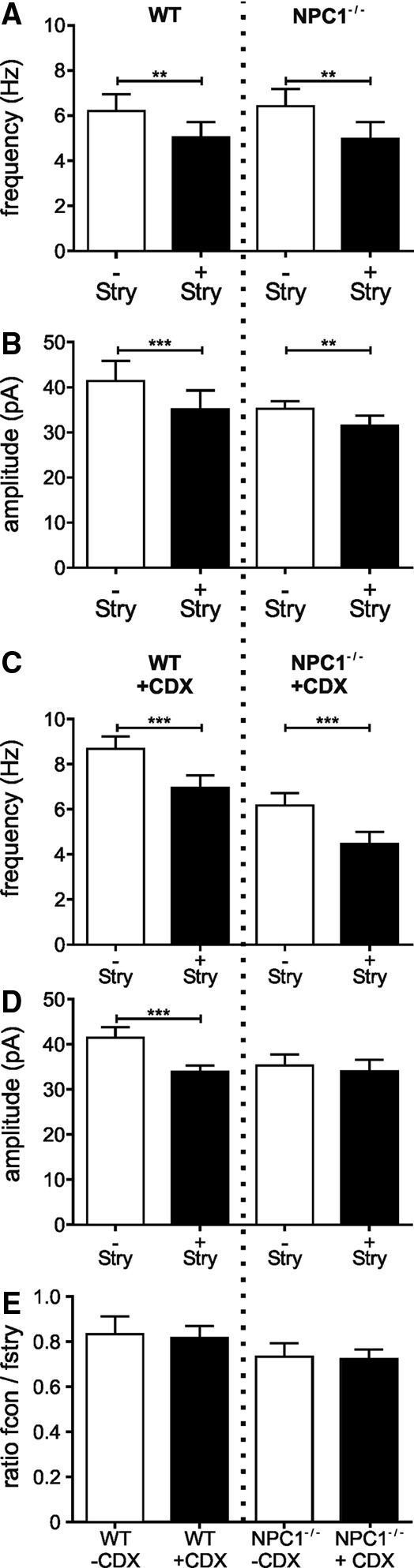
**(A)** Bar chart of the mean frequency of IPSCs in the absence (−Stry) and presence (+Stry) of strychnine. Strychnine reduced significantly the frequencies as well as the amplitudes in mice of both genotypes **(B)**. **(C)** Strychnine reduced the frequency in CDX-treated WT mice as well as in CDX-treated NPC1^−/−^ mice **(B)**, but only the amplitudes in WT mice were reduced **(D)**. The ratio fcon/fstry showed no differences between treated and untreated animals of both genotypes **(E)**.

Strychnine induced a significant reduction of the frequency in animals of both genotypes (WT: *f* = 5.0 ± 0.7 Hz, *n* = 22; NPC1^−/−^: *f* = 4.9 ± 0.7 Hz, *n* = 27; Fig. 2A) as well as a significant reduction of the amplitudes (WT: control 41.4 ± 4.5 pA, *n* = 22; Stry: 35.1 ± 4.1 pA, *n* = 22; NPC^−/−^: control 35.2 ± 1.7 pA, *n* = 27; Stry: 31.5 ± 2.2 pA, *n* = 27; Fig. 2B). Consistent with the results of untreated mice, the IPSCs were abolished by GBZ, where strychnine significantly reduced the frequency of the IPSCs in treated mice (WT/CDX: *f* = 6.9. ± 0.5 Hz, *n* = 17; NPC1^−/−^/CDX: *f* = 4.5 ± 0.7 Hz, *n* = 19; Fig. 2C). In contrast to untreated WT mice, in the presence of strychnine, a significant reduction of the amplitudes was observed in CDX-treated WT animals (WT/CDX: 33.9 ± 1.4 pA, *n* = 17) but not in CDX-treated NPC1^−/−^ mice (NPC1^−/−^/CDX: 34.1 ± 2.5 pA, *n* = 19; Fig. 2D). However, the calculation of the proportion of the frequency in the presence and the absence of strychnine (Fig. 2E) revealed no differences between untreated and CDX-treated animals, indicating that CDX most likely does not impair the glycinergic transmission.

## Discussion

Besides CDX, as a cholesterol-binding agent, several strategies for treatment of NPC1 are being under investigation, aiming at a reduction of lipid overload.^11,12^ The life span and the onset of neurodegenerative processes were clearly affected in NPC1^−/−^ mice treated with CDX, providing a promising intervention strategy for NPC1.^9,13^

Surprisingly, we found that CDX alters the inhibitory transmission in wild type, but not in NPC1^−/−^ mice. Actually, we expected an altered inhibitory synaptic transmission in the hippocampus of NPC1^−/−^ mice, as CA1 pyramidal cells receive input from multiple regions, and an alteration of pathways innervating the hippocampus was described for NPC1^−/−^ mice.^14^ Furthermore, an altered expression of glutamate and GABA transporters was found in the hippocampus of NPC1^−/−^ mice.^15^ Such alterations might lead to a hampered neurotransmitter level or neurotransmitter clearance, disturbing synaptic transmission. However, we did not reveal differences of GABAergic-IPSCs between both genotypes in control recordings as well as in recordings in the presence of the Gly-R antagonist strychnine. In accordance with other studies, we did not observe that IPSCs mediated by synaptically clustered Gly-Rs^16^ as in the hippocampus Gly-Rs act through extrasynaptically clustered receptors.^16,17^

Regarding the unexpected effect of CDX, we can only speculate about the underlying mechanism. Acting as an excipient and absorption enhancer,^18^ CDX can interact with lipid rafts,^19^ leading to altered synaptic clustering of receptors,^20,21^ or directly with ion channels, leading to altered channel properties,^22^ numerating only some actions of CDX.^18^ Our findings might be explained by an interaction of CDX with the machinery of the excitatory transmission, leading to increased frequencies of IPSCs as this is modulated by the activation of excitatory *N*-Methyl-d-aspartate receptors.^23^ Regarding the missing effect in NPC1^−/−^ mice, one can speculate that a typically elevated cholesterol level of NPC1-deficient cells changes the action of CDX. One feature of CDX is the ability to extract cholesterol, for example, from membranes in a dose-dependent manner. Up to a saturation of 65%, CDX depletes cholesterol from membranes, but with higher saturation it provides cholesterol.^24^ In addition, one has to consider rapid actions of CDX *in vitro*, like the induction of endolysosomal exocytosis within 30 min after administration.^25^ The amount of such rapidly released cholesterol might differ between WT- and NPC1-deficient animals leading to different interaction affecting the synaptic transmission.

## Conclusion

The observed effect of CDX in WT mice remains to be elucidated. However, our experiments add another aspect to the actions of CDX and might be of importance regarding adverse effects of intervention strategies for NPC1 based on compounds interacting with cholesterol.

## Abbreviations Used

NPC1: Niemann–Pick type C1 disease
CDX: 2-hydroxypropyl-b-cyclodextrin
WT: wild-type
IPSCs: inhibitory postsynaptic currents
Gly-Rs: glycine receptors
